# Handgrip strength to predict extubation outcome: a prospective multicenter trial

**DOI:** 10.1186/s13613-021-00932-3

**Published:** 2021-10-02

**Authors:** Guillaume Cottereau, Jonathan Messika, Bruno Megarbane, Laurent Guérin, Daniel da Silva, Caroline Bornstain, Matilde Santos, Jean-Damien Ricard, Benjamin Sztrymf

**Affiliations:** 1 AP-HP, Service de Rééducation Fonctionnelle et Kinésithérapie, Hôpital Antoine Béclère, 92140 Clamart, France; 2 AP-HP, Hôpital Louis Mourier, DMU ESPRIT, Service de Médecine Intensive Réanimation, Université de Paris, 92700 Colombes, France; 3 PHERE UMRS 1152, Université de Paris, 75018 Paris, France; 4 Réanimation Médicale et Toxicologique, Fédération de toxicologie, Hôpital Lariboisière, Université Paris-Diderot, Inserm UMRS 1144, 2, rue Ambroise-Paré, 75010 Paris, France; 5 AP-HP, Hôpitaux Universitaires Paris-Sud, Hôpital de Bicêtre, Service de Réanimation Médicale, 78, Rue du Général Leclerc, 94270 Le Kremlin-Bicêtre, France; 6 Faculté de Médecine Paris-Sud, Univ Paris-Sud, Inserm UMR_S 999, 94270 Le Kremlin-Bicêtre, France; 7 Réanimation Polyvalente, Hôpital Delafontaine, 93200 Saint-Denis, France; 8 Réanimation Polyvalente, Hôpital Intercommunal de Montfermeil, 93370 Montfermeil, France; 9 AP-HP, Service de réanimation polyvalente et surveillance continue, Hôpital Antoine Béclère, 157 rue de la porte de Triveaux, 92140 Clamart, France

**Keywords:** Handgrip, Intensive care unit-acquired weakness, Weaning, Extubation

## Abstract

**Background:**

ICU-acquired weakness (ICUAW) has been shown to be associated with prolonged duration of mechanical ventilation and extubation failure. It is usually assessed through Medical Research Council (MRC) score, a time-consuming score performed by physiotherapists. Handgrip strength (HG) can be monitored very easily at the bedside. It has been shown to be a reproducible and reliable marker of global muscular strength in critical care patients. We sought to test if muscular weakness, as assessed by handgrip strength, was associated with extubation outcome.

**Methods:**

Prospective multicenter trial over an 18 months period in six mixed ICUs. Adults receiving mechanical ventilation for at least 48 h were eligible. Just before weaning trial, HG, Maximal Inspiratory Pressure (MIP), Peak Cough Expiratory Flow (PCEF) and Medical Research Council (MRC) score were registered. The attending physicians were unaware of the tests results and weaning procedures were conducted according to guidelines. Occurrence of unscheduled reintubation, non-invasive ventilation (NIV) or high-flow nasal continuous oxygen (HFNC) because of respiratory failure within 7 days after extubation defined extubation failure. The main outcome was the link between HG and extubation outcome.

**Results:**

233 patients were included. Extubation failure occurred in 51 (22.5%) patients, 39 (17.2%) required reintubation. Handgrip strength was 12 [6–20] kg and 12 [8–20] kg, respectively, in extubation success and failure (*p* = 0.85). There was no association between extubation outcome and MRC score, MIP or PCEF. Handgrip strength was well correlated with MRC score (*r* = 0.718, *p* < 0.0001). ICU and hospital length of stay were significantly higher in the subset of patients harboring muscular weakness as defined by handgrip performed at the first weaning trial (respectively, 15 [10–25] days vs. 11 [7–17] days, *p* = 0.001 and 34 [19–66] days vs. 22 [15–43] days, *p* = 0.002).

**Conclusion:**

No association was found between handgrip strength and extubation outcome. Whether this was explained by the appropriateness of the tool in this specific setting, or by the precise impact of ICUAW on extubation outcome deserves to be further evaluated.

*Trial registration* Clinical Trials; NCT02946502, 10/27/2016, URL: https://clinicaltrials.gov/ct2/results?cond=&term=gripwean&cntry=&state=&city=&dist=

**Supplementary Information:**

The online version contains supplementary material available at 10.1186/s13613-021-00932-3.

## Background

The mechanical ventilation weaning process, including definite separation from the mechanical ventilator remains challenging in the intensive care unit (ICU). Despite a successful weaning trial, up to 20% of patients may experience post-extubation respiratory failure [1–3]. This subset of patients usually requires an unscheduled ventilatory support, and re-intubation in this setting has been associated with dismal prognosis [4, 5].

ICU-acquired weakness (AW) is the result of a combination of muscle and nerve injuries developed during ICU stay in 25 to 63% of mechanically ventilated patients [6, 7]. Pathophysiological mechanisms include bioenergetic failure, metabolic and microvascular injuries as consequences of critical illness [8]. ICUAW has been shown to delay liberation from mechanical ventilation and to increase ICU length of stay [9–11]. It may affect both peripheral and respiratory muscles, but conflicting results exist regarding the precise weight of these two types of muscle injuries on weaning outcome [12–14]. The Medical Research Council (MRC) Score is considered to be the gold standard to evaluate peripheral muscles’ strength. It is a bedside time-consuming test requiring an experienced physiotherapist [15]. Value of maximal handheld dynamometric strength measurement, referred as handgrip strength, has also been proven to be a reliable diagnostic tool, and a handgrip strength definition of ICUAW has been proposed [16]. Handgrip strength is a simple, non-invasive tool. It can be monitored very quickly and easily at the bedside [17]. It has been shown to display reliable and reproducible results [18]. It has been previously evidenced that severe limb weakness, as assessed through MRC, was associated with extubation outcome [19–21]. Two studies displayed conflicting results regarding the association between muscular strength, assessed with handgrip, and extubation outcome [22, 23]. Therefore, considering the major interest of diagnosing ICUAW to anticipate extubation failure, we conducted a multicenter prospective study aiming at testing the value of handgrip strength in predicting extubation outcome. We also sought to explore respiratory muscle strength on weaning outcome, using non-invasive tools.

## Methods

### Design and settings

We conducted a prospective multicenter trial over an 18 months period in 6 ICUs, from 4 university affiliated hospitals, and 2 general hospitals. Written informed consent was obtained from all patients or their next of kin. The protocol was approved by an institutional review board, the Comité de Protection des Personnes Paris Ile de France VII, according to French law. The study received no commercial support. This study was granted by the French Ministry of Health (PHRIP P150948). It was registered in ClinicalTrials.gov under the identifier NCT02946502.

### Population

Adult patients receiving mechanical ventilation for at least 48 h and meeting readiness-to-wean criteria according to international guidelines [24] were eligible. Patients were included unless they exhibited exclusion criteria. Exclusion criteria were: pre-existing rheumatologic, neurologic or orthopedic condition precluding the use of the handgrip, delirium as attested by the CAM-ICU [25], inability to deliver clear information to the patient (language barrier without interpreter for instance), pregnancy or participation to any other trial leading to modification of the usual weaning management.

### Study protocol

#### Handgrip strength testing

Trained physiotherapists assessed handgrip strength. Subjects were positioned as close to upright as possible, with the shoulders in neutral rotation at the subject’s side and elbow flexion of 90°. An adjustable handheld dynamometer (Jamar hydraulic hand dynamometer, Fred Sammons, Bolingbrook, Illinois) was used to obtain handgrip strength measurements. The dominant hand of each subject was tested. As recommended by the manufacturer, subjects were asked to hold the dynamometer at their maximum strength for 2–3 s. Examiners showed the subjects how to perform the test beforehand and encouraged them. The value of the highest handgrip strength after 3 consecutive attempts, separated by brief pauses (~ 30 s), was retained. As previously proposed, a handgrip strength of less than 11 kg in men and 8 kg in women defined muscular weakness [16].

#### Peak cough expiratory flow evaluation

Cough strength was evaluated through measurements of peak cough expiratory flow (PCEF). In that purpose, the proximal tip of the tracheal tube was linked to a spirometer (Asma 1, Vitalograph, Buckingham, England). The patient was then asked to take a deep inspiration through the spirometer (which allows air flow in inspiration without measuring the inspiratory flow) and to cough as strongly as possible. The highest peak cough expiratory flow after 3 consecutive attempts, separated by brief pauses (~ 30 s), was monitored. A single-use disposable tip was placed between the spirometer and the tracheal tube. The spirometer was cleaned as recommended by the manufacturer.

#### Maximal inspiratory pressure measurement

Inspiratory muscle strength was evaluated through the measurement of voluntary maximal inspiratory pressure (MIP). It was monitored only in patients connected to ventilators offering this option. Physiotherapists informed the patients that a brief occlusion of the inspiratory way was going to occur. The patients were then asked to inspire as strongly as possible. The highest inspiratory pressure after 3 consecutive attempts, separated by brief pauses (~ 30 s), was monitored.

#### MRC and arm abduction evaluation

Muscular testing was performed through measurement of the MRC score. As previously described, the muscle scale grades 6 groups of muscle power on a scale of 0 to 5 in relation to the maximum expected for that muscle. A score of less than 48 has been proven to indicate muscular weakness [26].

Physiotherapists performed a semi-quantitative evaluation of the dominant arm abduction: no movement, an angle from 0 to 45°, or more than 45° between the arm and the chest. This test was motivated by the old, barely documented [27] and ongoing belief that a patient able to perform a significant abduction of the arm would be able to be extubated.

### Experimental plan

The weaning protocol (encompassing decision to perform a weaning trial, and criteria evaluating success or failure of this trial as well as extubation failure criteria) followed international guidelines [22]. If readiness-to-wean conditions were met, patients were included unless they decline to participate or existence of an exclusion criterion. Included patients underwent evaluation of handgrip strength, MIP, peak cough expiratory flow, MRC and arm abduction as described above at each weaning trial. The attending physician was unaware of the results of these tests, and the weaning procedure was conducted according to guidelines. A 30-min to 2-h spontaneous breathing trial, whether t-tube breathing or low-level pressure support, was performed as recommended [22]. In case of success, patient was then extubated. In an attempt to decrease the rate of post-extubation respiratory failure in frail or old patients harboring chronic respiratory or cardiac impairment, prophylactic non-invasive ventilation (NIV) or high-flow nasal continuous oxygen (HFNC) could be performed as recommended [28, 29]. This decision was left at the attending physician’s discretion. In case of SBT failure, extubation was not performed and mechanical ventilation was resumed. Therefore, in such case, no association between the previously performed HG, MIP and PCEF could be tested. As performed in everyday care, a diagnostic workup was performed in order to find the cause of SBT failure. Another SBT was performed as soon as readiness-to-wean conditions were gathered again, on the following day whenever possible. Post-extubation respiratory failure was defined according to current guidelines: tachypneoa, tachycardia, clinical signs of respiratory muscle fatigue or increased inspiratory load, hypercapnia or hypoxemia [22]. The management of post-extubation respiratory failure was left at the attending physician’s discretion: mechanical ventilation could be resumed, or HFNC and/or NIV could be used as recue respiratory supports. Occurrence of unscheduled reintubation, NIV or HFNC because of post-extubation respiratory failure within 7 days after extubation defined extubation failure.

### Primary study endpoint

The main study endpoint was the association between handgrip strength and extubation outcome.

### Secondary study endpoints

Secondary study endpoints were the comparison of MIP, MRC, peak expiratory flow, arm abduction according to extubation outcome. ICU, hospital and 6 months survival were registered as well and compared according to muscular weakness as assessed by handgrip strength [16].

### Statistical analysis

Sample size was determined on the basis of our previous work showing a handgrip strength of 16 [7–23] kg in extubation success and 10 [5–18] kg in extubation failure [23]. A group of 30 patients experiencing extubation failures (and therefore 180 with extubation success when considering a failure rate of 15%) was required to demonstrate a significant difference between the aforementioned handgrip thresholds, with a 90% power and 5% alpha risk. To take into account *invalid data*, we anticipated to include 260 patients.

Patients’ characteristics are reported as numbers (percentages) for categorical data and as medians (25th–75th percentiles) for continuous data. Chi-square test was used to assess differences between groups for qualitative variable. Student’s t-test for independent samples or Mann–Whitney–Wilcoxon test was used to assess differences between two groups for quantitative variable. For example, handgrip as well as respiratory or muscular physiological variables were compared using Student’s *t*-test for independent samples or Mann–Whitney–Wilcoxon test between patients who were successfully extubated and those who failed extubation. Those tests were also used to compare demographic and physiologic variables between patients who succeeded or failed first weaning trial. Kruskal–Wallis test was used to assess difference between three groups. Associations between quantitative variables were analyzed with the Spearman correlation. SAS software version 9.4 was used for statistical analyses. P values below 0.05 were considered to denote statistical significance.

## Results

**Fig. 1:**
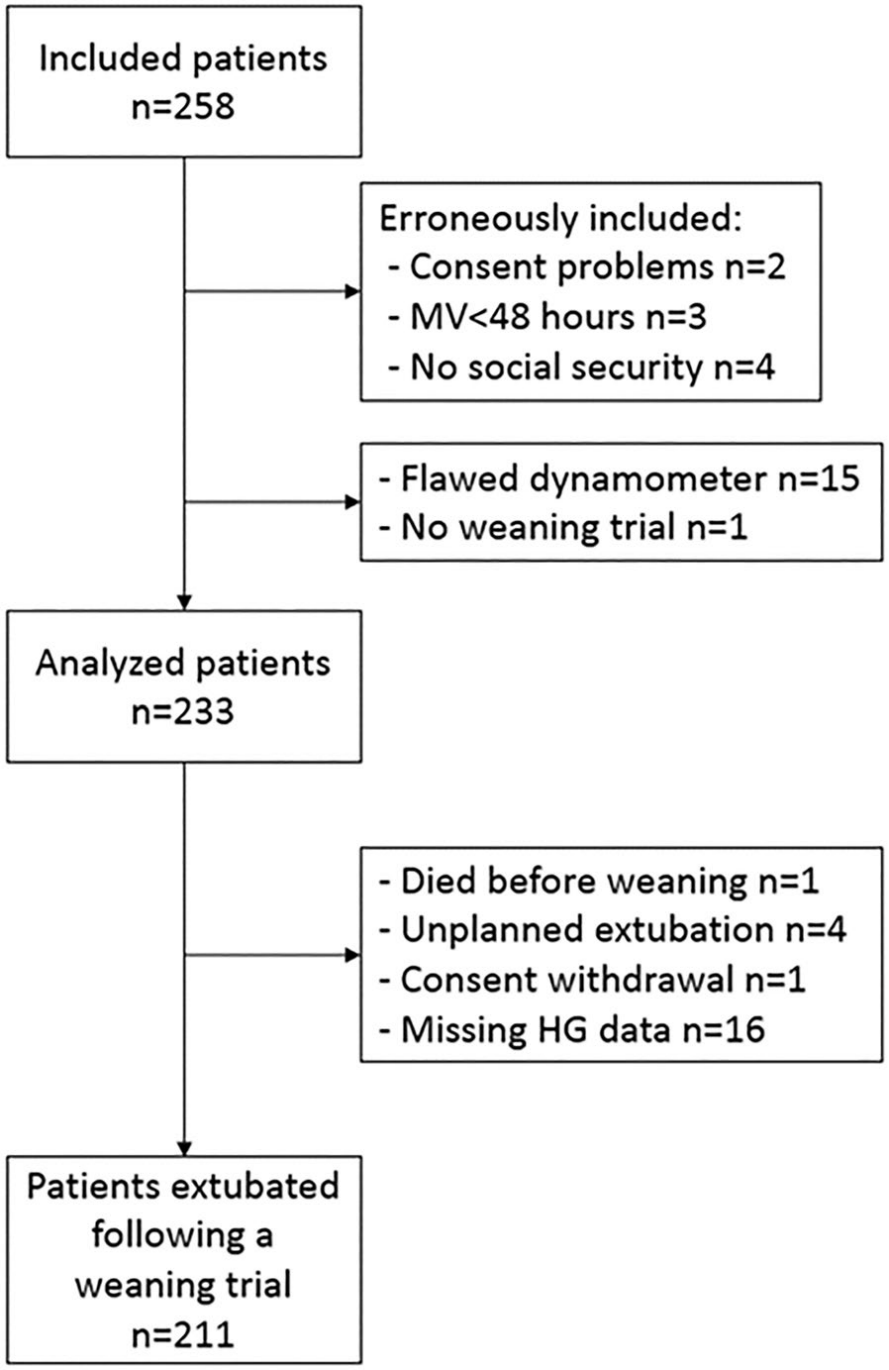
Flowchart. *MV* mechanical ventilation, *HG* handgrip

Overall, 233 patients were included (Fig. 1 and Additional file 1: Table S1). Their median age was 66 [53–75] years and 139 (59.6%) were men. Other main characteristics are displayed in Table 1. Based on current consensus [24], weaning was defined as simple, difficult and prolonged in 164 (70.4%), 49 (21%) and 18 (7.6%) patients, respectively. Prophylactic HFNC or NIV were provided following a planned extubation in 57 (25.1%) and 104 (45.8%) patients, respectively.

**Table 1:** Characteristics of the population

Variables	Population (*n* = 233)
Age (y)	66 [53–75]
Gender (M/F)	139/94
BMI (kg/m^2^)	28.9 ± 7.9
Dominant hand (R/L)	210/23
SAPS II	53 [40–67]
Baseline respiratory disease^a^ *n* (%)	74 (31.8)
COPD	41 (17.6)
Other	36 (15.4)
Baseline cardiac disease^a^ *n* (%)	88 (37.8)
LVF	45 (19.3)
Other	33 (14.2)
Reason for admission *n* (%)	
Shock	52 (22.3)
De novo ARF	66 (28.3)
Hypercapnic ARF	22 (9.4)
Neurologic failure	41 (17.6)
Post-operative	19 (8.2)
Other	33 (14.2)
Time spent under MV at inclusion (d)	6 [3–10]

*Y* years, *M* male, *F* female, *R* right, *L* left, *SAPS* Simplified Acute Physiology Score, *COPD* chronic obstructive pulmonary disease, *LVF* left ventricular failure, *ARF* acute respiratory failure, *MV* mechanical ventilation, *d* days

^a^Some patients harbored more than one baseline comorbidity making the whole sample inferior to the sum of both subgroups

### Extubation outcome

**Fig. 2:**
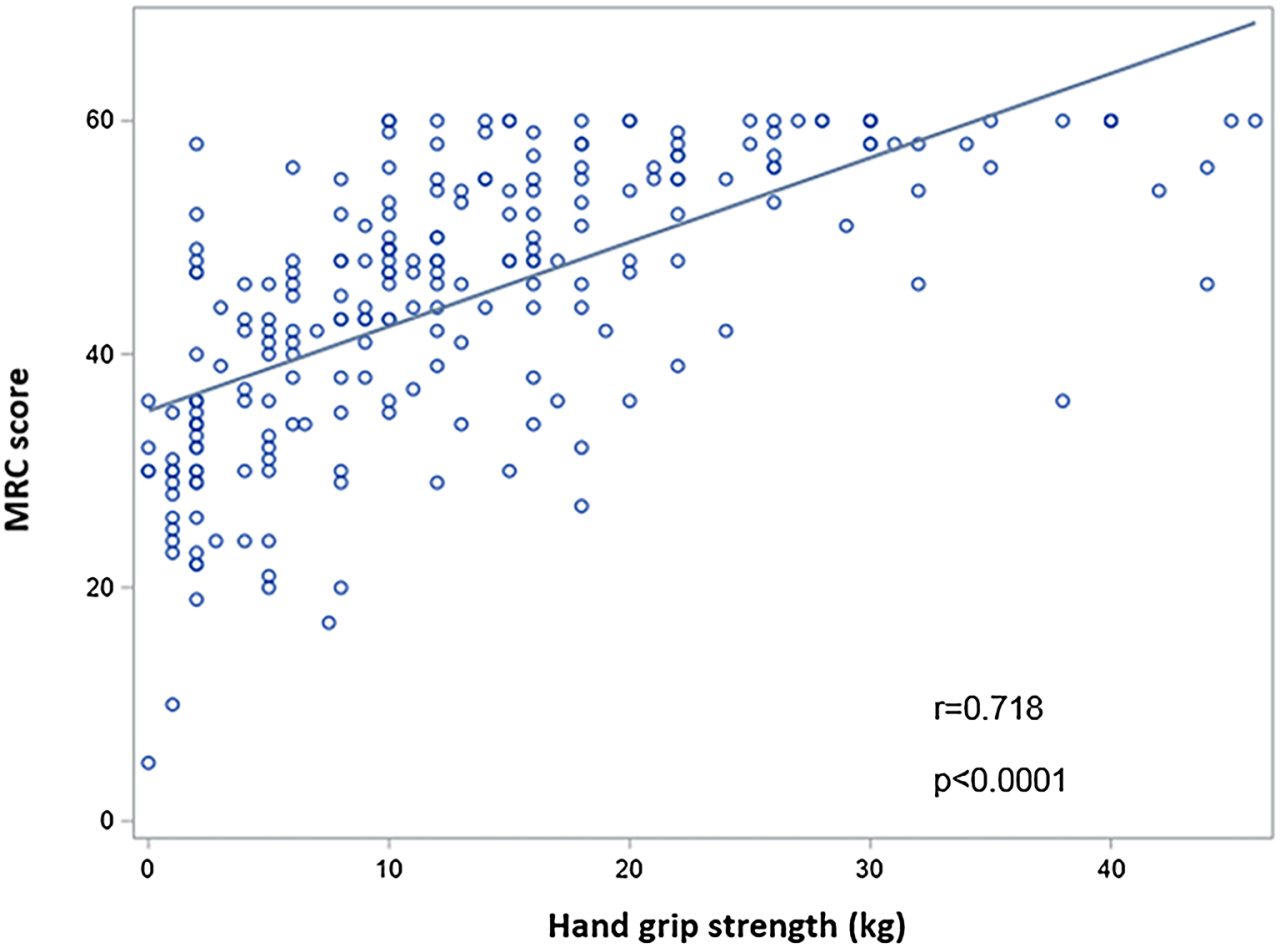
Association between handgrip strength and MRC score

Extubation failure occurred in 51 (22.5%) patients among whom 39 (17.2%) required reintubation (Additional file 1: Figure S1). Among the 27 patients who needed reintubation or who failed extubation, respectively, 22 (9.7%) and 5 (2.2%) underwent NIV and HFNC to treat post-extubation respiratory failure. Handgrip strength did not differ according to extubation outcome: 12 [6–20] kg and 12 [8–20] kg, respectively, in extubation success and failure (*p* = 0.85) (Table 2). Muscular weakness as defined by handgrip strength pointed to a very low predictive value with a sensitivity of 0.50, a specificity of 0.45 at the threshold of 14 kg (Additional file 1: Figure S2). MRC score, MRC score subgroup or dominant arm abduction angle did not differ between extubation success and extubation failure (Table 2). Handgrip strength was well correlated with MRC score (*r* = 0.718, *p* < 0.0001) (Fig. 2). The correlation remained significant in the subgroup of patients harboring muscular weakness with MRC < 48 (*r* = 0.520, *p* < 0.0001). Neither maximal inspiratory pressure, nor cough peak expiratory flow was associated with extubation outcome (Table 2). When looking only at reintubation as an endpoint, a trend toward a lower MRC score was found in reintubated patients (46 [34–50] vs. 47 [36–56]; *p* = 0.07).

**Table 2:** Muscular and respiratory tests according to extubation outcome

Variables	Extubation		*p*
	Success (*n* = 176)	Failure (*n* = 51)	
Handgrip strength (kg)	12 [6–20]	12 [8–20]	0.85
Muscular weakness *n* (%)	64 (38.6)	15 (33.3)	0.52
MRC score	48 [40–56]	47 [38–56]	0.67
MRC score subgroups *n* (%)			0.44
< 36	35 (21.1)	7 (15.6)	
36–47	44 (26.5)	16 (35.6)	
≥ 48	87 (52.1)	22 (48.9)	
Dominant arm abduction subgroups *n* (%)			0.82
0°	8 (4.8)	2 (4.4)	
0–45°	58 (34.9)	18 (40)	
45–90°	100 (60.2)	25 (55.6)	
Maximal inspiratory pressure (cmH_2_O)	31 [17, 19–41]	32 [19–45]	0.92
Peak expiratory cough flow (L/min)	65 [47–82]	61 [50–77]	0.80

Missing data *n* = 16 for handgrip, MRC score and dominant arm abduction (*n* = 10 and *n* = 6, respectively, in success and failure subgroups), *n* = 82 for maximal inspiratory pressure abduction (*n* = 62 and *n* = 20, respectively, in success and failure subgroups), *n* = 26 for peak expiratory cough flow (*n* = 17 and *n* = 9, respectively, in success and failure subgroups)

*MRC* Medical Research Council

Age was associated with extubation outcome (62.5 [53–73] years vs. 70 [58–78] years, respectively, in extubation success and failure, *p* = 0.025). There was a non-significant trend toward a higher incidence of baseline respiratory comorbidities in the subset of patients experiencing extubation failure (41.2% vs. 29.6%, *p* = 0.12) (Additional file 1: Table S2). Handgrip strength was associated with weaning difficulty with values of 12 [6–20] kg, and 8 [2–14] kg, respectively, in simple, and non-simple (i.e., difficult and prolonged) weaning subgroups of patients (*p* = 0.0012).

### Length of stay and survival

Overall ICU and hospital LOS (as defined by time lag from the day of admission to the day of discharge) were significantly higher in the subset of patients harboring muscular weakness as defined by handgrip strength performed at the first weaning trial (respectively, 15 [10–25] days vs. 11 [7–17] days, *p* = 0.001 and 34 [19–66] days vs. 22 [15–42] days, p = 0.002).

Regarding survival, 3 included patients died in ICU. Overall, 14 patients died in hospital, 8 (8.3%) and 6 (4.5%), respectively, in patients with and without muscular weakness (*p* = 0.24).

### Weaning trial outcome

At first weaning trial, age, sepsis at admission, baseline cardiac disease, MRC score and peak expiratory cough flow were associated with trial success. There was a non-significant trend toward a lower handgrip strength in the subset of patients experiencing a failure at first weaning trial (Table 3). A non-significant higher incidence of patients harboring a chronic baseline respiratory disease was evidenced as well (39.3% vs. 29.1%, *p* = 0.14).

**Table 3:** Characteristics of patients according to first weaning trial outcome

Variables	First weaning trial		*p*
	Success (*n* = 171)	Failure (*n* = 60)	
Age (y)	64 [52–74]	68 [58–77]	0.01
BMI (kg/m^2^)	28.2 [22.8–33]	27.9 [23.8–33.9]	0.89
Gender (M/F)	103/69	36/25	0.90
SAPS II	52 [40–65]	54 [45–72]	0.16
Sepsis *n* (%)	95 (55.2)	44 (72.1)	0.021
Baseline respiratory disease *n* (%)	50 (29.1)	24 (39.3)	0.14
Baseline cardiac disease *n* (%)	57 (33.1)	31 (50.8)	0.015
MRC score	47 [37–55]	43 [33–52]	0.028
MRC score subgroup *n* (%)			0.19
< 36	37 (21.6)	18 (30)	
36–47	49 (28.5)	20 (33.3)	
≥ 48	85 (49.7)	22 (36.7)	
Handgrip strength (kg)	12 [5–18]	8.5 [2.5–16]	0.057
Dominant arm abduction subgroup *n* (%)			0.47
0°	11 (6.4)	6 (10)	
0–45°	58 (33.9)	23 (38.3)	
45–90°	102 (59.7)	31 (51.7)	
Maximal inspiratory pressure (cmH_2_O)	30 [17, 20–41]	27 [16–37]	0.41
Peak expiratory cough flow (L/min)	62 [47–80]	55 [31–69]	0.006

*BMI* body mass index, *SAPS* Simplified Acute Physiology Score, *MRC* Medical Research Council

## Discussion

The main results of the present study are as follows: (i) muscular strength monitored by handgrip strength performed just before weaning trial did not help to prognosticate extubation outcome; (ii) as already evidenced, we found that muscular strength monitored by handgrip strength predicted difficult weaning; (iii) peak cough expiratory flow predicted failure of weaning trial, there was a trend toward a lower handgrip strength in the subgroup of patients experiencing failure at first weaning trial.

Extubation is a complex process involving both hemodynamic and respiratory modifications [22, 30]. A potential imbalance between the global respiratory load and the ability to overcome such load can eventually occur, with subsequent post-extubation respiratory failure and its associated dismal prognosis in case of reintubation. It has been previously shown that ICUAW was associated with weaning difficulties [7–11], but few studies directly investigated extubation outcome [19–21, 23]. Our study tried to shed light on a daily question in ICU: “When added to a weaning trial, could a simple evaluation of muscular strength help to prognosticate extubation outcome?” The present study failed to show an association between extubation outcome and muscular strength, as monitored by handgrip dynamometry.

The lack of prognostic performance of handgrip testing might have various explanations. One of the hypothesis is that handheld dynamometer is not the appropriate tool in this setting. It has been found to be reliable, with a good inter-observer reliability mainly outside the ICU [31–33], with less thorough evaluation in critically ill patients [16, 34, 35]. It has been previously suggested that muscular strength evaluation through handheld dynamometry lacks precision in the low range of strength [17, 36, 37]. In previous studies, despite a handgrip strength of zero, some patients harbored a near-normal manual muscle testing through MRC [35, 38], which was found in a subset of patients in the present work as well. Furthermore, ICUAW is generally more pronounced in proximal muscles [6, 8]. Though this work did not aim at testing the association between MRC and extubation outcome, neither proximal nor distal muscles testing showed any association with extubation outcome in the present cohort. Therefore, though a good correlation has been found between MRC and handgrip in the present work, as in previous studies, it may be hypothesized that handheld dynamometer was not suitable for monitoring the ICUAW consequences on extubation outcome in our subset of patients. We would like to underline that the present study is in agreement with previous works showing an association between muscular weakness and overall delay in liberation from mechanical ventilation [9–11, 23, 24].

Our study failed to evidence any association between cough strength and extubation success. Cough strength is a major determinant of bronchial secretions clearance. When monitored using PCEF, cough strength has been shown to be associated with extubation success in many studies [19, 39–41]. One first reason could be the volitional pattern of the test, raising the possibility of a decreased strength in both subgroup of patients. Another potential explanation is that weaning protocol followed usual practice in all aspects of the process. Physicians in charge in the investigating centres often perform a simple evaluation of cough strength at the bedside before allowing a patient to begin a weaning trial. It might be hypothesized that physicians in charge postponed the weaning trial in some patients because of a clinically estimated insufficient cough strength. Monitoring the peak cough expiratory flow appears to be a reliable surrogate to the evaluation of the ability to clear bronchial secretions. Nevertheless, we would like to underline that we did not monitor the amount of bronchial secretions, which might further increase the value of cough strength in our study. Last, though PCEF was not related to extubation outcome, it was associated to weaning trial outcome. Therefore, patients with low PCEF more often failed the weaning trial and were not extubated, limiting its impact on extubation outcome. A similar phenomenon was observed for handgrip strength with a trend toward a lower value in patients experiencing a weaning trial failure. Such patients were not extubated, and the potential link with extubation failure could not be determined.

### Limitations

In spite of its prospective design, and strictly conducted protocol, our study has several limitations that deserve to be underlined. First, we excluded the first 15 included patients in one centre because of a logistic pitfall. The dynamometer available in this centre always indicated that handgrip strength was equal to zero, even when participating physiotherapists tested it themselves. Provided that handgrip was the main endpoint of the study, we estimated mandatory to exclude these patients, but recognize that it was unscheduled and might have biased the results. Second, the respiratory tests used in the present study, though simple and non-invasive, might depend on the level of participation of the patients. This could have biased our evaluations. Nevertheless, gold standards tests to assess respiratory muscles, namely bilateral phrenic nerve stimulation and diaphragmatic echography were not available in our study, as they were not routinely performed in all investigating centres within the study period. Third, it has to be noted that time to peak force generation in critical care patients might be as long as 6 s [42]. We allowed up to three seconds for patients to develop a maximal grip strength, but may have interrupted the test too quickly to register the maximal strength in some patients. Fourth, imbalances in the recruitment rate between centers may have biased the results despite the standardization in the weaning process. Fifth, though weaning process followed the international guidelines throughout the study, individual’s estimation of insufficient cough or muscular strength might have led to postpone the weaning trials, potentially decreasing the value of the test. Last, a recent study showed that ICU-acquired weakness was independently associated with reintubation in the ICU [19]. To demonstrate this finding, the authors pooled two previous studies showing that ICU-acquired weakness was associated with reintubation using univariate analysis although it did not remain significantly associated with reintubation after multivariable analysis. Indeed, neither of the studies showed that ICU-acquired weakness was independently associated with reintubation after multivariate analysis probably because each one was underpowered. Therefore, despite a rigorous evaluation of the sample of patients, one cannot exclude that the present study might also be somewhat underpowered.

## Conclusion

Altogether, no association was found between handgrip strength and extubation outcome. Whether this was explained by the study design, the appropriateness of the tool in this specific setting, or by the precise impact of ICUAW on extubation outcome deserves to be further evaluated.

## Supplementary Information

**Additional file 1: Table S1.** Inclusions distribution among participating centers. **Table S2.** Baseline characteristics according to extubation outcome. **Figure S1.** Weaning flowchart. **Figure S2.** Receiver operating characteristics analysis testing acuity of handgrip to predict extubation outcome.

## Data Availability

All data generated or analyzed during this study are included in this published article [and its additional information files].

## Abbreviations

ICU: Intensive care unit
ICUAW: Intensive care unit-acquired weakness
HFNC: High-flow nasal continuous oxygen
HG: Handgrip strength
LOS: Length of stay
MIP: Maximal inspiratory pressure
NIV: Non-invasive ventilation
PCEF: Peak cough expiratory flow

## References

[1] Jaber S, Quintard H, Cinotti R (2018). Risk factors and outcomes for airway failure versus non-airway failure in the intensive care unit: a multicenter observational study of 1514 extubation procedures. Crit Care.

[2] Penuelas O, Frutos-Vivar F, Fernández C (2011). Characteristics and outcomes of ventilated patients according to time to liberation from mechanical ventilation. Am J Respir Crit Care Med.

[3] Thille AW, Harrois A, Schortgen F (2011). Outcomes of extubation failure in medical intensive care unit patients. Crit Care Med.

[4] Epstein SK, Ciubotaru RL, Wong JB (1997). Effect of failed extubation on the outcome of mechanical ventilation. Chest.

[5] Frutos-Vivar F, Esteban A, Apezteguia C (2011). Outcome of reintubated patients after scheduled extubation. J Crit Care.

[6] Hermans G, Van den Berghe G (2015). Clinical review: intensive care unit acquired weakness. Crit Care.

[7] Sharshar T, Bastuji-Garin S, Stevens RD (2009). Presence and severity of intensive care unit-acquired paresis at time of awakening are associated with increased intensive care unit and hospital mortality. Crit Care Med.

[8] Hermans G, Horn J. Intensive care unit acquired weakness. In: Wijdicks EFM, Kramer AH, editors. Handbook of clinical neurology, vol. 141 (3rd series) Critical Care Neurology, Part II. 2017. p. 532–43. 10.1016/B978-0-444-63599-0.00029-6 28190434

[9] De Jonghe B, Bastuji-Garin S, Sharshar T (2004). Does ICU-acquired paresis lengthen weaning from mechanical ventilation?. Intensive Care Med.

[10] Garnacho-Montero J, Amaya-Villar R, Garcia-Garmendia JL (2005). Effect of critical illness polyneuropathy on the withdrawal from mechanical ventilation and the length of stay in septic patients. Crit Care Med.

[11] De Jonghe B, Bastuji-Garin S, Durand MC (2007). Respiratory weakness is associated with limb weakness and delayed weaning in critical illness. Crit Care Med.

[12] Dres M, Dube BP, Mayaux J (2017). Coexistence and impact of limb muscle and diaphragm weakness at time of liberation from mechanical ventilation in medical intensive care unit patients. Am J Respir Crit Care Med.

[13] Medrinal C, Prieur G, Frenoy É (2017). Is overlap of respiratory and limb muscle weakness at weaning from mechanical ventilation associated with poorer outcomes?. Intensive Care Med.

[14] Dres M, Jung B, Molinari N (2019). Respective contribution of intensive care unit-acquired limb muscle and severe diaphragm weakness on weaning outcome and mortality: a post hoc analysis of two cohorts. Crit Care.

[15] Stevens RD, Marshall SA, Cornblath DR (2009). A framework for diagnosing and classifying intensive care unit-acquired weakness. Crit Care Med.

[16] Ali NA, O’Brien JM, Hoffmann SP (2008). Acquired weakness, handgrip strength, and mortality in critically ill patients. Am J Respir Crit Care Med.

[17] Bittner EA, Martyn JA, George E (2009). Measurement of muscle strength in the intensive care unit. Crit Care Med.

[18] Bragança RD, Ravetti CG, Barreto L (2019). Use of handgrip dynamometry for diagnosis and prognosis assessment of intensive care unit acquired weakness: a prospective study. Heart Lung.

[19] Thille AW, Boissier F, Muller M (2020). Role of ICU-acquired weakness on extubation outcome among patients at high risk of reintubation. Crit Care.

[20] Jeong BH, Nam J, Ko MG (2018). Impact of limb weakness on extubation failure after planned extubation in medical patients. Respirology.

[21] Dres M, Similowski T, Goligher EC (2021). Dyspnea and respiratory muscles ultrasound to predict extubation failure. Eur Respir J.

[22] Cottereau G, Dres M, Avenel A (2015). Handgrip strength predicts difficult weaning but not extubation failure in mechanically ventilated subjects. Respir care.

[23] Saiphoklang N, Tepwimonpetkun C (2020). Interest of handgrip strength to predict outcome in mechanically ventilated patients. Heart Lung.

[24] Boles JM, Bion J, Connors A (2007). Weaning from mechanical ventilation. Eur Respir J.

[25] Ely EW, Inouye SK, Bernard GR (2001). Delirium in mechanically ventilated patients: validity and reliability of the confusion assessment method for the intensive care unit (CAM-ICU). JAMA.

[26] De Jonghe B, Sharshar T, Lefaucheur J-P (2002). Paresis acquired in the intensive care unit: a prospective multicenter study. JAMA.

[27] De Beer CR, van Rooijen AJ, Pretorius JP (2018). Muscle strength and endurance to predict successful extubation in mechanically ventilated patients: a pilot study evaluating the utility of upper-limb muscle strength and ergometry. S Afr J Crit Care.

[28] Rochwerg B, Einav S, Chaudhuri D (2020). The role for high flow nasal cannula as a respiratory support strategy in adults: a clinical practice guideline. Intensive Care Med.

[29] Rochwerg B, Brochard L, Elliott MW (2017). Official ERS/ATS clinical practice guidelines: non-invasive ventilation for acute respiratory failure. Eur Respir J.

[30] Thille AW, Richard JCM, Brochard L (2013). The decision to extubate in the ICU. Am J Respir Crit Care Med.

[31] Peolsson A, Hedlund R, Oberg B (2001). Intra- and inter-tester reliability and reference values for hand strength. J Rehabil Med.

[32] Savva C, Giakas G, Efstathiou M, Karagiannis C (2014). Test-retest reliability of handgrip strength measurement using a hydraulic hand dynamometer in patients with cervical radiculopathy. J Manipulative Physiol Ther.

[33] Sasaki H, Kasagi F, Yamada M, Fujita S (2007). Grip strength predicts cause specific mortality in middle-aged and elderly persons. Am J Med.

[34] Vanpee G, Segers J, Van Mechelen H (2011). The interobserver agreement of handheld dynamometry for muscle strength assessment in critically ill patients. Crit Care Med.

[35] Parry SM, Berney S, Granger CL (2015). A new two-tier strength assessment approach to the diagnosis of weakness in intensive care: an observational study. Crit Care.

[36] Bohannon RW (2001). Measuring knee extensor muscle strength. Am J Phys Med Rehabil.

[37] Wadsworth CT, Krishnan R, Sear M (1987). Intrarater reliability of manual muscle testing and hand-held dynametric muscle testing. Phys Ther.

[38] Lee JJ, Waak K, Grosse-Sundrup M (2012). Global muscle strength but not grip strength predicts mortality and length of stay in a general population in a surgical intensive care unit. Phys Ther.

[39] Beuret P, Roux C, Auclair A (2009). Interest of an objective evaluation of cough during weaning from mechanical ventilation. Intensive Care Med.

[40] Smina M, Salam A, Khamiees M (2003). Cough peak flows and extubation outcomes. Chest.

[41] Almeida CM, Lopes AJ, Guimarães FS (2020). Cough peak flow to predict the extubation outcome: comparison between three cough stimulation methods. Can J Respir Ther.

[42] Baldwin CE, Paratz JD, Bersten AD (2013). Muscle strength assessment in critically ill patients with handheld dynamometry: an investigation of reliability, minimal detectable change, and time to peak force generation. J Crit Care.

