# Supplementary material for: Handgrip strength to predict extubation outcome: a prospective multicenter trial
Source: Ann Intensive Care. 2021 Oct 2;11:144. doi: 10.1186/s13613-021-00932-3 (PMC8487340; doi:10.1186/s13613-021-00932-3)
Supplement: Supplementary file 1 — Additional file 1: Table S1. Inclusions distribution among participating centers. Table S2. Baseline characteristics according to extubation outcome. Figure S1. Weaning flowchart. Figure S2. Receiver operating characteristics analysis testing acuity of handgrip to predict extubation outcome. [file 13613_2021_932_MOESM1_ESM.docx]

**Additional files**

Table S1: Inclusions distribution among participating centers

|  | Inclusions n (%) |
| --- | --- |
| Antoine Beclere hospital | 80 (34.3) |
| Louis Mourier hospital | 65 (27.9) |
| Bicêtre Hospital | 39 (16.8) |
| Le Raincy Montfermeil hospital | 4 (1.7) |
| Lariboisière hospital | 34 (14.6) |
| Saint Denis - Delafontaine hospital | 11 (4.7) |

Figure S1: weaning flow chart


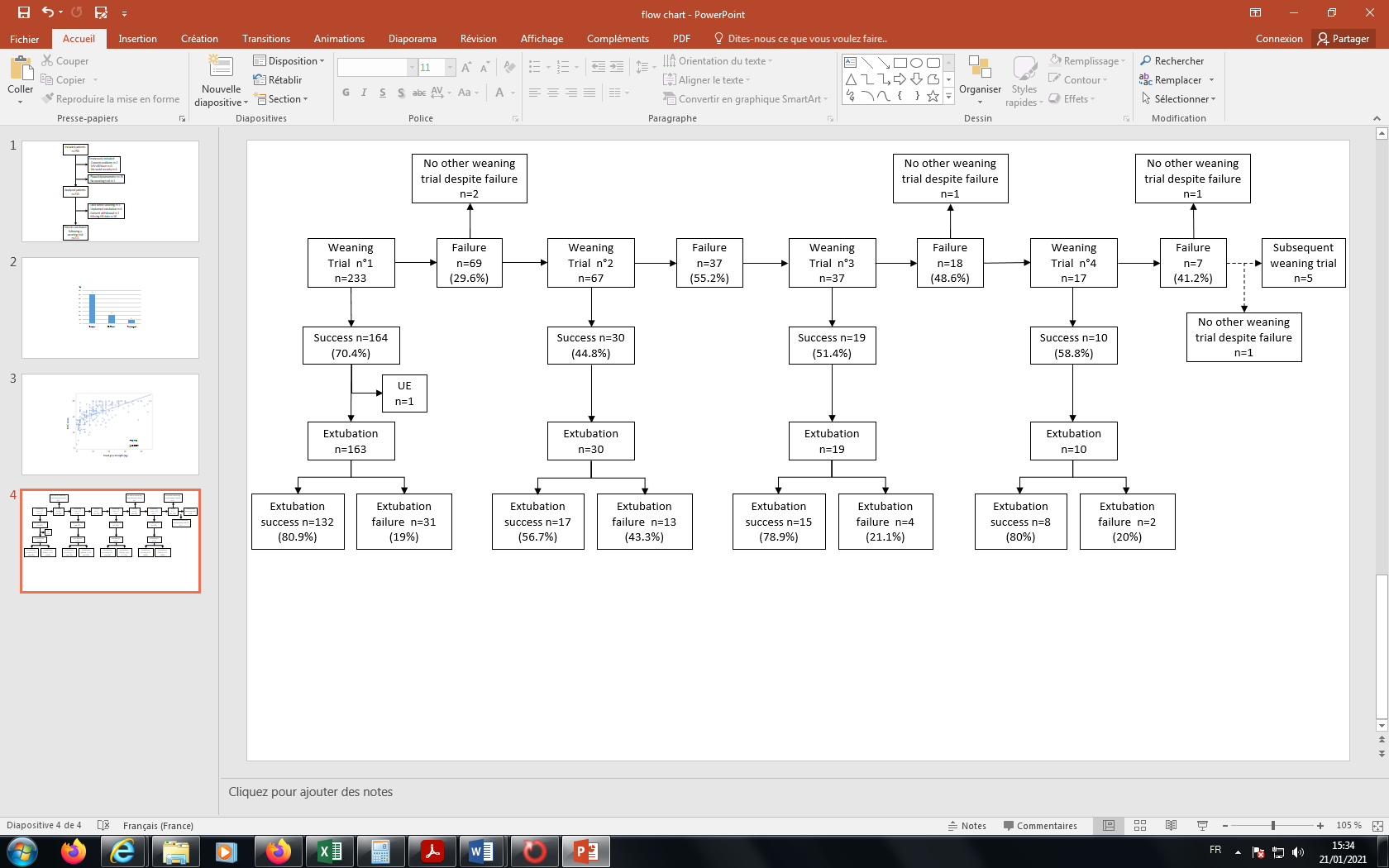


UE= unplanned extubation

Figure S2: receiver operating characteristics analysis testing acuity of handgrip to predict extubation outcome.


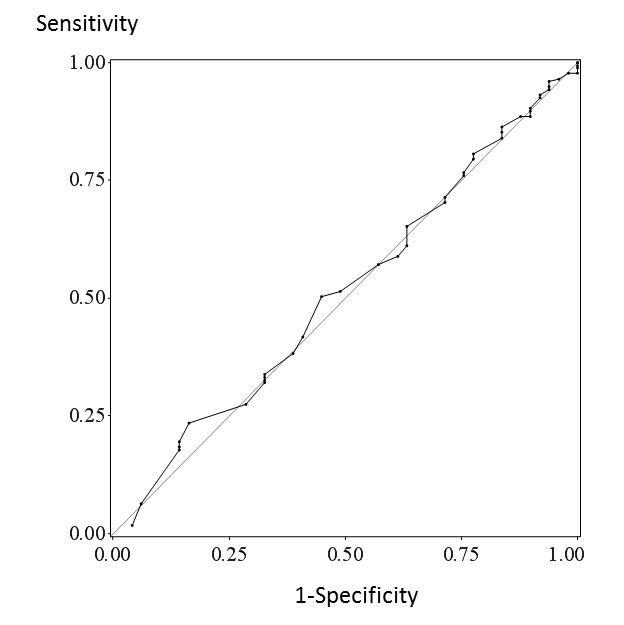


At the threshold of 14 kg, Sensitivity was 0.42 and specificity 0.63.

Table S2: baseline characteristics according to extubation outcome

|  | Extubation | | p |
| --- | --- | --- | --- |
|  | Success (n=176) | Failure (n=51) |  |
| Age (y) | 62.5 [53-73] | 70 [58-78] | 0.025 |
| Gender (M/F) | 106/70 | 30/21 | 0.86 |
| BMI (Kg/m2) | 29.2 [23.2-33.4] | 27.1 [21.6-30.2] | 0.18 |
| Dominant hand (R/L) | 159/17 | 45/6 | 0.67 |
| Baseline respiratory disease n(%) | 52 (29.6) | 21 (41.2) | 0.12 |
| Baseline cardiac disease n(%) | 70 (39.7) | 16 (31.4) | 0.28 |
